# Telomerase expression and activity in the immortal cnidarian Hydra

**DOI:** 10.3389/fcell.2026.1893030

**Published:** 2026-08-18

**Authors:** Dmitry A. Skvortsov, Maria P. Rubtsova, Vladimir S. Dolzhnikov, Andrey G. Zaraisky, Fedor M. Eroshkin

**Affiliations:** 1 Chemistry Department, Lomonosov Moscow State University, Moscow, Russia; 2 Shemyakin-Ovchinnikov Institute of Bioorganic Chemistry, Russian Academy of Sciences, Moscow, Russia; 3 Koltzov Institute of Developmental Biology, Russian Academy of Sciences, Moscow, Russia

**Keywords:** Hydra, immortality, senescence, telomerase, TERT

## Abstract

Hydra is one of the few known immortal animals, but the cellular and molecular mechanisms underlying this phenomenon remain largely unclear. Telomere lengthening is recognized as one of the factors contributing to enhanced proliferative potential at the cellular level. Telomerase activity is known to support both cellular and organismal longevity. In this study, we characterized the telomerase of *Hydra vulgaris*. The expression pattern of the telomerase catalytic subunit (*hyTERT*) mRNA correlates with regions exhibiting high proliferative potential and remains unchanged during budding or regeneration. These findings were further corroborated by measurements of telomerase activity. Our data suggest that telomerase may play a role in the immortality of *Hydra*.

## 1 Introduction

Most living eumetazoan animals are mortal. A fundamental mechanism underlying cellular and organismal aging in many species is the progressive shortening of telomeres—protective nucleoprotein structures at chromosome ends. The finite replicative lifespan of somatic cells, described by Hayflick and Moorhead (Hayflick and Moorhead, 1961), was theoretically linked to telomere attrition by Olovnikov (Olovnikov, 1973). The discovery of telomerase, a ribonucleoprotein complex with reverse transcriptase activity that synthesizes telomeric repeats, provided a molecular explanation for the unlimited proliferative potential of germlines, stem cells, and most cancer cells (Greider and Blackburn, 1985). Its two core components are a catalytic protein subunit, telomerase reverse transcriptase (TERT), and an RNA template (TR) (Harrington and McPhail, 1997).

In humans, telomerase is not expressed in most somatic tissues and is active primarily in germ cells, stem cells, and certain rapidly renewing tissues such as blood or intestinal epithelium. Moreover, telomerase is highly upregulated in the majority of malignant tumors (Kim et al., 1994). Induction of TERT reverse transcriptase expression is widely used for cell immortalization, including applications in basic research and regenerative medicine (Sutyagina and Bellin, 2023; Shitova and Alpeeva, 2024). Comparative studies indicate that telomere dynamics correlate with lifespan, with slower shortening rates in long-lived species (Dantzer and Fletcher, 2015). Recent cross-species analyses, however, reveal a more complex picture, suggesting that the relationship between telomere maintenance strategies (telomerase activity vs. alternative lengthening) and longevity is not strictly deterministic (Gomes and Ryder, 2011). Nevertheless, it remains a prevailing hypothesis that biologically immortal organisms must possess robust mechanisms for telomere maintenance (Schaible et al., 2015). Supporting this, sustained telomerase activity in somatic tissues has been documented in non-senescent invertebrate models such as planarians (Tan et al., 2012) and long-lived bivalves (Gruber and Schaible, 2014).

Importantly, several taxa, including some hydras, planarians, and rotifers, have been reported to exhibit negligible or undetectable senescence (Austad, 2009). In a four-year-long experiment, Daniel Martínez demonstrated that the *Hydra* did not show measurable age-related increase in mortality or decline in reproductive capacity, suggesting an absence of detectable senescence over the observation period (Martinez, 1998). While initially met with scrutiny (Estep, 2010), these findings were corroborated by population studies confirming constant mortality and fertility rates (Schaible et al., 2015). Consequently, *Hydra* has emerged as a key model for studying the basis of biological immortality and the evolution of aging (Nebel and Bosch, 2012; Bellantuono and Bridge, 2015; Tomczyk and Fischer, 2015; He and Bosch, 2022).

Basal metazoans, including cnidarians like *Hydra*, possess the canonical vertebrate-type telomeric repeat (TTAGGG)n (Traut et al., 2007). While telomerase activity has been detected in the gonads of other cnidarians and ctenophores (Traut et al., 2007), and somatic activity reported in the jellyfish *Cassiopea* (Ojimi et al., 2009) and during life cycle reversal in *Turritopsis* (Matsumoto et al., 2019), previous attempts to detect it in *Hydra* were unsuccessful, likely due to methodological limitations (Traut et al., 2007). Thus, despite its status as a paradigmatic non-senescent organism, the question of telomerase activity in Hydra remains open.

## 2 Methods

### 2.1 *Hydra* culture and sampling strategy

All experiments were performed on the asexual strain of *H. vulgaris* (former *H. magnipapillata*) strain 105. Animals were starved 1–2 days prior to any experiments. For each biological replicate, 15 animals cut with a microscalpel under a binocular microscope, by using an eye micro-knife, into three body regions: head (tentacles + hypostome), middle body (gastric region), and foot (peduncle + basal disc). We chose this approach to sampling based on the differences in the proliferative potential and cellular differentiation of different parts of the hydra body. Tissues from 15 animals were pooled for RNA isolation or protein extraction (for TRAP method), and this was repeated in at least 3 independent biological replicates.

### 2.2 Bioinformatic approaches

Evolutionary analysis by the Maximum Likelihood method was conducted in MEGA12 software (Kumar and Stecher, 2024). The phylogeny was inferred using the Maximum Likelihood method and Jones and Taylor (1992) model (Jones and Taylor, 1992) of amino acid substitutions and the tree with the highest log likelihood (−88 101.63) is shown. The percentage of replicate trees in which the associated taxa clustered together (500 replicates) is shown next to the branches (Felsenstein, 1985). The initial tree for the heuristic search was selected by choosing the tree with the superior log-likelihood between a Neighbor-Joining (NJ) tree (Saitou and Nei, 1987) and a Maximum Parsimony (MP) tree. The NJ tree was generated using a matrix of pairwise distances computed using the Jones-Taylor-Thornton model (Jones and Taylor, 1992). The MP tree had the shortest length among 10 MP tree searches, each performed with a randomly generated starting tree. The analytical procedure encompassed 35 amino acid sequences with 1 804 positions in the final dataset.

### 2.3 Whole-mount *in situ* hybridization

*In situ* hybridization was performed as described (Bode and Lengfeld, 2008). Fragment corresponding to *hyTERT* mRNA (714 b.p.) was amplified with primers CTT​CAA​CCA​ATC​ATA​AGC​AGC​A and TGA​CAC​ACT​CCA​TAC​CAT​CT, cloned into pAL-TA vector (Evrogen). Sense- and antisense probes were synthesized from the linearized plasmid. Hybridization was performed with both sense- and antisense probes at concentration 0.3 mkg/mL. Staining was performed with NBT/BCIP as a substrate. Negative (sense- and no probe) controls were also performed.

### 2.4 RT-qPCR

Total RNA was isolated using NucleoSpin® RNA XS kit (Macherey-Nagel), RNA integrity was assessed by agarose gel electrophoresis, RNA concentration and purity were measured using the Implen spectrophotometer (A_260_/A_280_ ratios between 1.9 and 2.1, A_260_/A_230_ > 1.9). All samples with acceptable quality were used for cDNA synthesis. cDNA was synthesized with MMLV RT kit (Evrogen). Real-time PCR performed using qPCRmix-HS SYBR (Evrogen) on DTprime amplificator (DNA-Technology) according to manufacturer’s recommendations. *Hydra EFα* and *GAPDH* homologs were used as reference, as their mRNA level was constant in our experiments (data not shown). Primers used are given in Table 1.

**TABLE 1:** Primers used for RT-qPCR.

Gene	Primer	Sequence (5'→3′)	Product size (bp)	PCR efficiency
hyEFα	Forward	GCT​TCA​TTC​AAA​GCC​CAG​GTT​A	96	2,02
	Reverse	AGC​AAT​GTG​AGC​AGT​GTG​AC		
hyGAPDH	Forward	ATT​GCC​TTG​CTC​CTC​TTG​CT	134	1,97
	Reverse	CCC​ATC​ACG​CCA​TTC​CTT​CA		
hyTERT (set 1)	Forward	TGC​AAT​TTG​AAG​GTT​TGA​GCA​TTA	135	1,95
	Reverse	GCT​ATC​TTT​GAT​ATG​TTC​CCC​TA		
hyTERT (set 2)	Forward Reverse	CTT​CAA​CCA​ATC​ATA​AGC​AGC​A	98	1,98
		CGA​ACA​ATA​GGA​ATT​AGA​AAG​TCA		

Two independent primer sets for *hyTERT* were used to confirm the specificity of the qPCR results. The qPCR assay reliably detected *hyTERT* mRNA in all body regions with Ct values between 26 and 34 cycles. No-template controls and no-reverse-transcriptase controls were negative in all runs. Agarose gel electrophoresis analysis of final PCR products confirmed a single specific band for each primer pair.

To evaluate the efficiency (E) of qPCR, we performed qPCR with four fivefold dilutions of cDNA templates. The averages of Ct data of each dilution from three replicates of qPCR with each pair of primers were used to determine the slope and to calculate the E value.

(E = 1/5−slope). The PCR data were imported into Microsoft Excel and analyzed using the ΔΔCt method. The geometric mean of expression of two reference housekeeping genes, *Hydra EFα* and *GAPDH*, was used for the normalization of the target gene expression values. For each gene, we performed at least 3 independent experiments. The one-way ANOVA followed by Tukey’s HSD *post hoc* test for comparisons among the body regions (head, body, foot, bud) was used (Supplementary Table S1).

### 2.5 Telomerase activity assay

*H.vulgaris* fragments and whole animals were frozen in liquid nitrogen and milled in MicroDismembrator U, then suspended in lysis buffer (10 mM Tris-HCl and 10 mM HEPES-KOH, pH 7.5, 1.0 mM MgCl2, 1 mM EGTA, 5 mM β-mercaptoethanol, 5% glycerol, 0.5% CHAPS, 0.1 mM PMSF) and five stroke in Dounce homogenizer (pestle B) were performed. The amount of frozen powder and buffer was adjusted to final protein concentration in extract 0.2–0.7 mg/mL. The samples were incubated for 30 min on ice, centrifuged for 10 min at 4° C for 15,000 g, and the supernatant solution was collected. The extract was aliquoted and frozen in liquid nitrogen. Telomerase activity was measured using the method described by Kim et al. (1994). Telomerase activity was normalized by protein quantity. Total protein was measured using the Bradford assay calibrated with BSA; samples were diluted to the linear range and measured in duplicate. Telomerase activity in Hydra was measured using an extract containing 0.5 μg of total protein. Because 2 µg per 24 µL of reaction caused overload of the TRAP assay which look as inhibition of reaction, whereas up to 0.5 µg per 24 µL there were no significant inhibition.

To control the specificity RNase treatment was used. 30 μL of extract were mixed with 3 µL of Rnase A solution (10 mg/mL) and incubated 20 min 37 C, that cause over 1000x decrease of activity. Untreated samples result in classical “ladder” of DNA fragments in gel-based version of TRAP assay (one experiment during extraction conditions optimization, data not shown).

The telomerase polymerase reaction was carried out with 24 μL of 1,2x master mix (1x mix contains 1X TRAP-buffer (1X TRAP-buffer: 20 mM HEPES-KOH pH 8.3, 1,5 mM MgCl2, 63 mM KCl, 1 mM EGTA, 0,1 mg/mL BSA, 0,005% v/v Tween-20), 20 pmol of dNTPs, 10 pmol of oligonucleotide TS (AAT​CCG​TCG​AGC​AGA​GTT) and 4 μL of extract. The reaction mixture was incubated for 30 min at 25 °C. Thereafter 1.5 units of Taq-DNA polymerase (“Helicon”), 10 pmol of oligonucleotide ACX (CGC​GGC​TTA​CCC​TTA​CCC​TTA​CCC​TTA​CC) and Sybr Green I to 0.2x final concentration in the mixture (together 2 μL volume) were added to reaction in ice. Real time PCR was carried out on the device CFX-96 for 35 s at 94 °C, 35 s at 50 °C, 90 s at 72 °C (30 cycles of thermal cycler Mastercycler (“Bio-Rad”)).

Each TRAP experiment was done in technical triplicate with the same extract and median was used for further quantification. Four independent replica of Hydra samples and its fragments preparations in different days was used. Mean and standard deviation were used for calculation. The one-way ANOVA followed by Tukey’s HSD *post hoc* test for comparisons among the body regions (head, body, foot, bud) was used (Supplementary Table S2).

## 3 Results

Telomerase RNA (TR) sequence shows very little evolutionary conservation, and therefore we were unable to identify it reliably in the *Hydra* genome using a standard bioinformatic search. In contrast, the *TERT* gene is evolutionarily conserved and can be easily identified in the *Hydra* genome (gene ID: 124819364). Hereafter, we refer to it as *hyTERT*.

**FIGURE 1:**
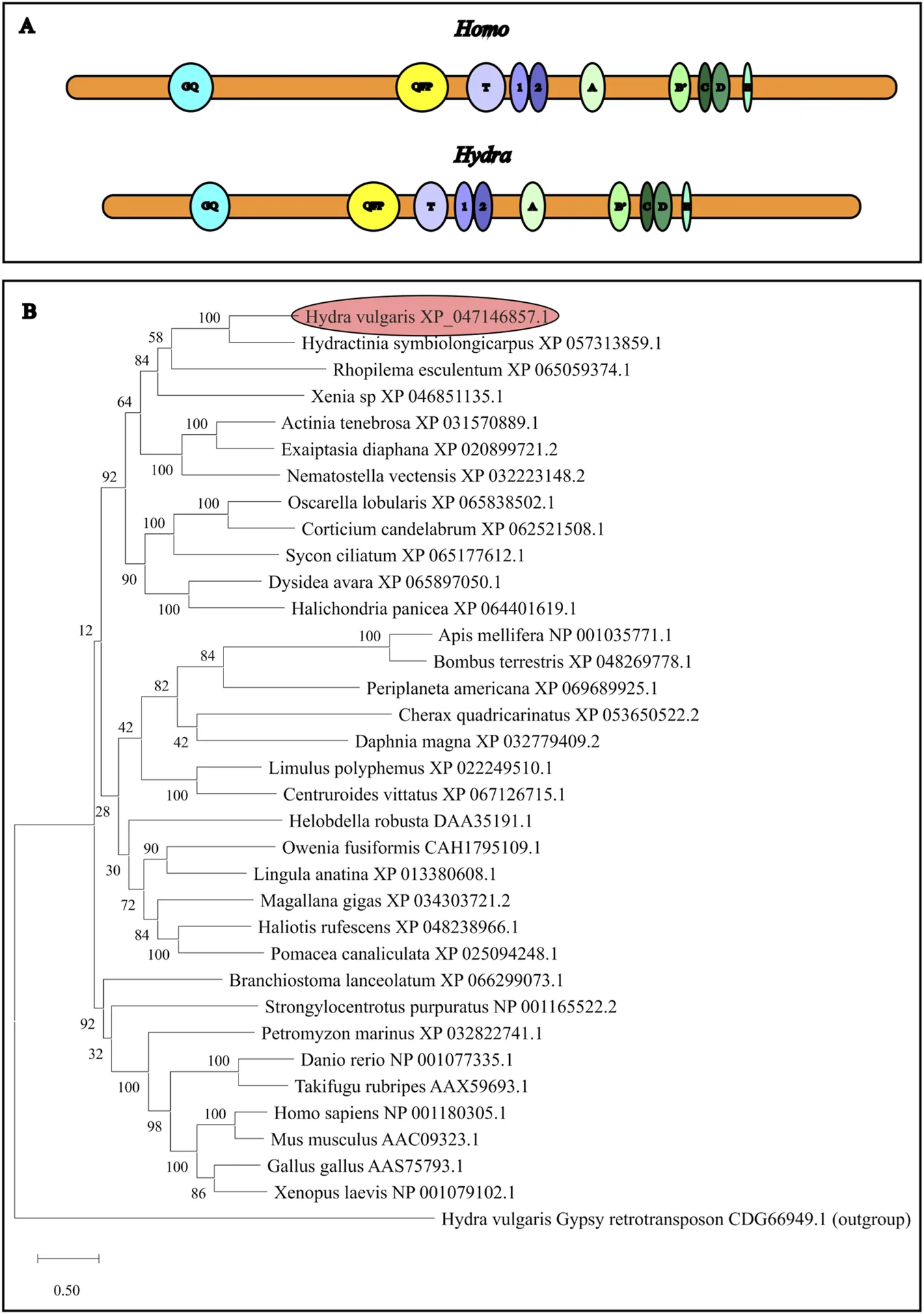
**(A)** Conserved motifs of *Homo* and *Hydra* TERT proteins. Motifs are marked in concordance with (Tan et al., 2012; Bryan and Sperger, 1998). **(B)** Phylogenetic tree of TERT proteins.

**FIGURE 2:**
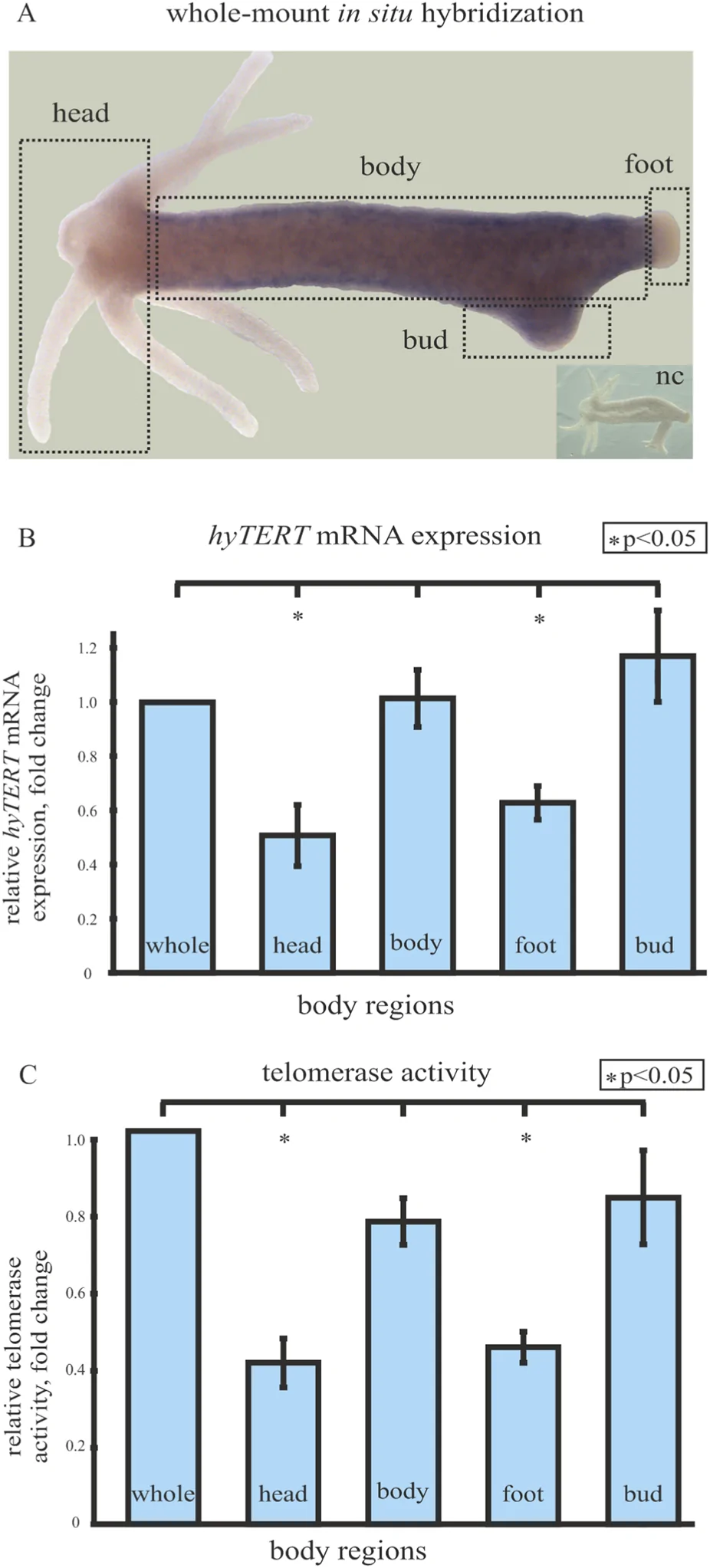
Distribution of the telomerase in *Hydra*. **(A)** Expression pattern of *hyTERT* mRNA in *Hydra*. nc–negative control (sense probe). **(B)** *hyTERT* mRNA expression in different parts of *Hydra* body, according to RT-qPCR data. mRNA level in whole animal was taken as 1. Bars indicate the standard deviation. Asterisks indicate significant differences from whole body value. **(C)** Telomerase activity in different parts of *Hydra* body. Telomerase activity in HEK293T cell line was taken as 1. Bars indicate the standard deviation. Asterisks indicate significant differences from whole body value.

Protein sequence alignment indicates that, compared to the human TERT protein, the *Hydra* protein is of comparable length and contains all the characteristic conserved motifs (Figure 1A). The phylogenetic position of hyTERT protein generally corresponds to the widely accepted tree of life (Figure 1B). To investigate the expression of the *hyTERT* gene, we analyzed the spatial distribution of hyTERT mRNA in *Hydra* using conventional whole-mount *in situ* hybridization. The expression pattern of hyTERT is diffuse, with a maximum signal in the middle of the body (Figure 2A), a region of the body characterized by active cell proliferation (see Discussion). This result was also confirmed by quantitative RT–PCR performed on RNA isolated from different regions of the *Hydra* body (Figure 2B).

To analyze which cell types express *hyTERT*, we used the *Hydra* single-cell database (Siebert and Farrell, 2019). We found that, consistent with our experimental data, *hyTERT* mRNA is expressed at a moderate level in most cell types, including ectodermal and endodermal epithelial cells and interstitial stem cells (Supplementary Figure S1). We also tested whether *hyTERT* mRNA expression changes during head regeneration after decapitation, but did not detect any significant differences (Supplementary Figure S2).

To test whether the *Hydra* body parts expressing *hyTERT* exhibit telomerase activity, and whether this activity correlates with mRNA levels, we measured telomerase activity in whole *Hydra* polyps. The fact that *Hydra* telomeres are identical to mammalian ones (Traut et al., 2007) allowed us to use the conventional TRAP assay. The telomerase activity level was quite high, comparable to that observed in the telomerase-positive immortalized human cell line HEK293T (used for normalization in Figure 2C), and several orders of magnitude higher than that in human peripheral white blood cells (Strazhesko and Tkacheva, 2016). Then we measured telomerase activity in different body regions of *Hydra*. It correlated well with *hyTERT* mRNA expression: the activity was high in the middle of the body and low in the head and foot regions (Figure 2C).

## 4 Discussion

In the present study, we demonstrate that: (i) *hyTERT* mRNA and telomerase activity in *Hydra vulgaris* are preferentially localized to the middle body region, an area characterized by active cell proliferation; (ii) *hyTERT* mRNA is detectable across a broad range of cell types, including epithelial cells; and (iii) *hyTERT* mRNA levels do not change significantly during asexual reproduction (budding) or regeneration. These findings are particularly relevant given that mid-body region of *Hydra* is known to harbor continuously proliferating populations of both interstitial stem cells and somatic epithelial cells. It is well established that *Hydra* polyps exhibit continuous tissue dynamics: cells proliferate intensively in the mid-body (Campbell, 1967), migrate toward the periphery (tentacles and foot), differentiate into various cell types, and are eventually shed at the extremities (Holstein and Hobmayer, 1991). In the tentacles and foot, cells become arrested in the G2 phase of the cell cycle (Dubel and Hoffmeister, 1987). The observed spatial correspondence between *hyTERT* expression and these proliferative zones (Supplementary Figure S3) is consistent with the hypothesis that telomerase activity may be linked to the maintenance of cell turnover in *Hydra*; however, the constitutive nature of this association, rather than an inducible response, represents one of the most notable aspects of our findings.

An important implication of our data is that telomerase activity in *Hydra* appears to be constitutively maintained at levels sufficient to support continuous tissue renewal, rather than being upregulated in response to regenerative demand. The observation that *hyTERT* mRNA levels do not increase during budding or regeneration is fully consistent with this interpretation: the enzyme is already present in proliferative zones under basal conditions, suggesting that telomerase activity is an integral component of constitutive tissue homeostasis. In this view, telomerase does not act as a externally inducible factor but rather as a steady-state contributor to the lifelong cell turnover that characterizes *Hydra* physiology. This interpretation does not exclude the possibility that telomerase also contributes to long-term maintenance of tissue integrity, but it emphasizes that the primary correlate of telomerase activity in *Hydra* is continuous proliferation rather than regenerative activation.

We next consider our data in the context of the existing, albeit currently fragmented, literature on telomere and telomerase biology in cnidarians. On one hand, telomerase activity has been detected in the jellyfish *Cassiopea*, with no significant differences observed among life cycle stages (Ojimi et al., 2009). That study represented the first report of telomerase activity in somatic (non germline) cells of a non bilaterian animal. This observation is noteworthy because the asexual polyp stage exhibits high longevity (up to 7 years), whereas the sexual medusa stage has a short lifespan. The authors proposed that this difference may be attributable to the presence of multipotent interstitial cells and a high regenerative capacity. Similarly, in the colonial coral *Galaxea*, telomere length remains unchanged across different life cycle stages (Tsuta and Hidaka, 2013). On the other hand, in the colonial coral *Acropora*, telomeres are longest in sperm and shortest in adult colonies (Tsuta et al., 2014), indicating that telomerase is active primarily in the germline—a pattern reminiscent of that observed in most mortal organisms, including mammals. In contrast, the hydrozoan *Turritopsis* provides a particularly instructive example, given its well documented capacity for life cycle reversal and the associated phenomenon of rejuvenation (Piraino et al., 1996). This species represents another potentially immortal organism and serves as a valuable model for aging research (Liu et al., 2023). Transcriptomic analyses across distinct life cycle stages of *Turritopsis* have revealed that telomerase components are upregulated during the rejuvenation process, accompanied by coordinated changes in transposable element regulation, DNA repair pathways, and the suppression of specific cell signaling cascades. These findings underscore the potential importance of telomerase activity in sustaining immortality (Matsumoto et al., 2019). Taken together, these studies illustrate considerable diversity in telomerase regulation across cnidarians, ranging from germline-restricted activity to constitutive somatic expression.

Whether the constitutively active telomerase in Hydra reflects an ancestral condition or a derived trait remains an open question. Given the currently limited data on telomerase activity across different life cycle stages in Cnidaria, one may suppose that the ancestral condition resembled that of other metazoans, namely, constitutive telomerase activity restricted largely to the germline. Its activation in somatic cells or at non germline life cycle stages may therefore have arisen secondarily and independently in lineages such as *Hydra* and *Turritopsis*. However, this hypothesis remains speculative, and a more systematic survey of telomerase activity across a wider phylogenetic range of cnidarians will be required to test such evolutionary scenarios.

In summary, our results demonstrate a close association between constitutive telomerase activity and proliferative tissues in *Hydra*. The data establish that telomerase is present in somatic cells and is spatially correlated with regions of active cell turnover, but they do not directly address whether this activity is functionally required for the sustained proliferative capacity or the apparent lack of senescence in this organism. Further experiments, including direct perturbation of telomerase function, will help to determine whether the observed activity is indeed necessary for tissue homeostasis, stem-cell maintenance, or other long-term physiological characteristics of *Hydra*.

## Data Availability

The raw data supporting the conclusions of this article will be made available by the authors, without undue reservation.

## Supplementary material

Supplementary Figure S1
**(A)** Distribution of *hyTERT* mRNA in different cell types of *Hydra* according to the single cell database (Siebert and Farrell, 2019). (https://portals.broadinstitute.org/single_cell/study/stem-cell-differentiation-trajectories-in-hydra-resolved-at-single-cell-resolution). *hyTERT* ID is g23858.t1. **(B)** Distribution of *hyWnt3* mRNA (ID g28064.t1), as an examlpe of very localized expression.

Supplementary Figure S2 h*yTERT* mRNA expression during regeneration after decapitation, according to qRT-PCR data. As a control were taken whole animals, mRNA level was taken as 1. Bars indicate the standard deviation.

Supplementary Figure S3 Distribution of *hyTERT* mRNA staining (lower curve, our data), and number of mitotic cells (middle and upper curves, according to Campbell, 1967-II, Fig. 3b) along the *Hydra* body axe.
