# Supplementary material for: Telomerase expression and activity in the immortal cnidarian Hydra
Source: Front Cell Dev Biol. 2026 Aug 18;14:1893030. doi: 10.3389/fcell.2026.1893030 (PMC13529943; doi:10.3389/fcell.2026.1893030)
Supplement: Supplementary file 1 [file Supplementaryfile1.docx]

**Supplementary Material**

**Supplementary Table S1. Tukey's HSD, hyTERT mRNA level (RT-qPCR method)**

| **pair** | **mean values difference** | **q** | **p** | **significant** |
| --- | --- | --- | --- | --- |
| Head — Trunk | -0,465 | 4,613 | < 0,05 | yes |
| Head — Foot | -0,032 | 0,322 | > 0,05 | no |
| Head — Bud | -0,517 | 5,134 | < 0,05 | yes |
| Head — whole | -0,492 | 4,886 | < 0,05 | yes |
| Trunk — Foot | 0,433 | 4,291 | > 0,05 | no |
| Trunk — Bud | -0,052 | 0,521 | > 0,05 | no |
| Trunk — whole | -0,027 | 0,273 | > 0,05 | no |
| Foot — Bud | -0,485 | 4,811 | < 0,05 | yes |
| Foot — whole | -0,46 | 4,563 | < 0,05 | yes |
| Bud — whole | 0,025 | 0,248 | > 0,05 | no |

**Supplementary Table S2. Tukey's HSD, telomerase activity (TRAP method)**

| **pair** | **mean values difference** | **q** | **p** | **significant** |
| --- | --- | --- | --- | --- |
| head — body | -0,41 | 5,082 | < 0,05 | yes |
| head — foot | -0,04 | 0,496 | > 0,05 | no |
| head — bud | -0,485 | 6,012 | < 0,01 | yes |
| head — whole | -0,564 | 6,986 | < 0,01 | yes |
| body — foot | 0,37 | 4,586 | < 0,05 | yes |
| body — bud | -0,075 | 0,93 | > 0,05 | no |
| body — whole | -0,154 | 1,904 | > 0,05 | no |
| foot — bud | -0,445 | 5,516 | < 0,05 | yes |
| foot — whole | -0,524 | 6,49 | < 0,01 | yes |
| bud — whole | -0,079 | 0,975 | > 0,05 | no |

**Supplementary Methods:** The distribution of *in situ* hybridization staining density was analyzed in the free ImageJ program using the plot profile function.
